# Is Improvement of Innovation Efficiency Conducive to Haze Governance? Empirical Evidence from 283 Chinese Cities

**DOI:** 10.3390/ijerph17176095

**Published:** 2020-08-21

**Authors:** Fei Fan, Dailin Cao, Ning Ma

**Affiliations:** 1 Institute of Regional and Urban-Rural Development, Wuhan University, Wuhan 430072, China; ffan@whu.edu.cn (F.F.); 2019106320001@whu.edu.cn (D.C.); 2 Institute of Central China Development, Wuhan University, Wuhan 430072, China; 3 School of Urban and Environment, Central China Normal University, Wuhan 430079, China

**Keywords:** innovation efficiency, haze pollution, threshold regression, mediation effect

## Abstract

In recent years, haze pollution has had a wide impact in China. This research systematically studies the influence mechanism of haze pollution from a new perspective of urban innovation efficiency. We use a generalised space two-stage least squares method to analyse the correlation between urban innovation efficiency and haze pollution. The periodic and regional influences of urban innovation efficiency on haze pollution is explored using a threshold regression model. Through the mediating effect model, we accurately identify the transmission mechanism of urban innovation efficiency affecting haze pollution. The results show a significant inverted ‘U’ relationship between improvement of urban innovation efficiency and haze pollution. The regional innovation activities of innovative cities differ greatly from those of non-innovative cities. The effect of innovation efficiency improvement in innovative cities on haze governance is better than that of non-innovative pilot cities. In eastern cities with a higher level of economic development, the improvement of innovation efficiency has a stronger impact on haze governance. Industrial structure and population agglomeration have a mediating effect on the impact of urban innovation efficiency on haze pollution, providing directions for the rational formulation and effective implementation of haze governance policies in China, as well as in other countries.

## 1. Introduction

In recent years, haze pollution in China has become increasingly serious with the rapid development of urbanisation and industrialisation. It has occurred at a high frequency and has had a wide range of impacts, the governance of which has proven to be difficult. As such, there is an urgent need to control haze pollution. In the context of China’s coal-dominated energy structure, the management of haze is a scientific and systematic issue that requires cooperation between both the government and enterprises. On the one hand, the government intensifies environmental regulations and forces enterprises to upgrade their technologies. This fundamentally changes the mode of economic growth from the pursuit of scale and speed to improved efficiency. On the other hand, it is necessary for responsible enterprises to take the lead in terms of social responsibility, introduction of advanced technology, and promotion of industrial upgrading. Innovation now plays an increasingly prominent role in haze governance. Only by constantly promoting technological progress can manufacturers realise chained emission reduction from clean production to terminal control as soon as possible. Most of the existing analyses on the influencing factors of haze governance only focus on economic impacts such as urbanisation [1], industrial structure [2], and fiscal expenditure [3]. Few scholars have focused on the relationship between innovation and haze governance, especially the impact of improvement in urban innovation efficiency (UIE) on the latter.

Innovation efficiency is an important indicator for measuring the level of innovation [4,5]. An interesting phenomenon in China is that regions with high innovation efficiency and high levels of haze pollution overlap to a large extent. China’s PM_2.5_ (fine particulate matter) high-emission regions are concentrated in the Beijing-Tianjin-Hebei and Yangtze River Delta urban agglomerations and surrounding areas, including Beijing, Tianjin, Shanghai, Jiangsu, Zhejiang, and other regions [2,6]. Similarly, the Yangtze River Delta, including Shanghai, Jiangsu, and Zhejiang provinces, and the Beijing-Tianjin-Hebei region, represented by Beijing, Tianjin, and other cities, are also agglomerations with high urban innovation efficiency [7]. Is this phenomenon an inevitable result of the process of economic development? What is the mechanism of its occurrence? Does innovation efficiency promote or mitigate haze pollution? Is the performance of different cities the same? How can we effectively achieve the ‘win-win’ goal of innovation development and haze governance based on a city’s economic development level? These are all urgent questions for academic circles to answer.

In view of this, this study uses data envelopment analysis (DEA) to measure the innovation efficiency of 283 prefecture-level cities in China from 2012 to 2016. The PM_2.5_ concentration is used to refer to the degree of haze pollution. Through generalised spatial two-stage least squares (GS2SLS), which can control both spatial spill over effects and endogeneity, this study conducts an empirical analysis of the influence of innovation efficiency on haze pollution. The marginal contribution of this research is threefold: (1) A systematic study is conducted on the influence mechanism of haze pollution from a new perspective—that of improving the efficiency of urban innovation. Considering that the core spatial carrier of China’s national innovation system is the city, 283 prefecture-level cities in China were considered as the research object. (2) We adopt a threshold regression model and use night-time light as the threshold variable. The staged and regional impact of UIE on haze pollution under different economic development levels is considered from a non-linear perspective. (3) According to the mediating effect test principle [8], we construct a mediating effect model composed of three regression equations to accurately identify the transmission mechanism through which UIE affects haze pollution. This is done to provide empirical support for the rational formulation and effective implementation of haze governance policies in China, as well as in other countries and regions.

## 2. Literature Review and Theoretical Hypotheses

At present, the purpose of most academic research is to explore the impact of technological progress on environmental pollution. Scholars have formed two diametrically opposed views based on this relationship: One is that technological progress has increased environmental pollution [9,10], while the other is that it has reduced environmental pollution [11,12]. It is generally believed that the relationship between innovation and environmental pollution is linear, ignoring the stage characteristics of innovation on environmental pollution, especially haze pollution. The impact of innovation on environmental pollution shows a trend of intensifying and then mitigating [13]. This is because in the initial stage of innovation, companies are more focused on developing production-oriented technologies at the expense of resources and the environment [10]. Because of the excessive pursuit of business value and economic growth by companies and governments, initial innovations are often solely focused on generating economic value, ignoring any environmental pollution problems caused by production [14]. Thus, increased research and development (R&D) investment causes environmental quality to decline due to the ‘squeeze out’ effect of the polluting manufacturing sector. The government then gradually realises the problem of environmental pollution and begins to adopt pollution emission restrictions and R&D subsidies for the clean energy sector. Investment in R&D gradually flows to the clean input sector, and the environmental pollution situation gradually improves [15]. Thus, the impact of an improvement in UIE on haze pollution is not a simple linear relationship; it may show an inverted ‘U’ trend of ‘positive followed by negative’. Based on the above views, we put forward the first theoretical hypothesis:

**Hypothesis** **1** **(H1).** *There is a nonlinear inverted ‘U’ relationship between UIE and haze pollution*.

Cities are the centre of regional economic and social development. They serve as the most important base for national economic output where various innovative elements and resources gather [7]. The development of cities has a major impact on the overall development of regions and countries. In order to give full play to the city’s core role in promoting independent innovation and accelerating the transformation of its economic development mode, China began to implement a pilot project of building an innovative city in 2008. The scope of the pilot has since continuously expanded. Innovative cities refer to those that mainly rely on innovation factors, such as science and technology, knowledge, manpower, culture, and systems, to drive its development. Such cities have high-end radiation and play a leading role for other regions. The innovation efficiency of innovative cities differs greatly from non-innovative cities, creating a large gap in the role of haze governance. With the in-depth advancement of the pilot project for innovative cities by the Chinese government, their innovation resources have continued to increase due to investment. The innovation factor agglomeration also continues to increase. Accordingly, the second hypothesis of this study is proposed:

**Hypothesis** **2** **(H2).** *There are regional differences in the effect of UIE improvement on haze governance, and the effect of UIE improvement in innovative pilot cities on haze governance is superior to non-pilot cities*.

As the economy develops, the intensity of government environmental regulations continues to increase, and the market welcomes more environmentally friendly technologies. In response to government requirements and market demand, companies invest more money in green innovation, which is conducive to environmental governance [12]. In addition, there are some economic factors that may influence the green investment strategy of enterprises, for example, our findings show that a longer relationship with the main bank fosters firms’ involvement in green investment strategies in order to reduce their environmental impact [16]. At the same time, based on the findings of Lin et al. [17], environmental regulation has a significant impact on the progress of green technology, showing a U-shaped trend where it first inhibits progress, then promotes it. That is, when the level of economic development is low and the intensity of environmental regulation is relatively strong, it cannot effectively promote the green technological progress of enterprises. If both the level of economic development and the intensity of environmental regulation increases, the effect of such regulations in promoting green technological progress improves. Therefore, due to the difference in the level of regional economic development, regional innovation activities are not the same. Likewise, the impact of innovation on the environment also varies. This indicates that in the mechanism of innovation’s effect on haze pollution, the level of economic development may play an important role and affect the position of the ‘inflection point’ in the relationship between the two. There may also be a threshold value of UIE improvement to mitigate the impact of haze pollution. That is, when the economic level is low, an improvement in UIE cannot reduce haze pollution, but it plays a restraining role on haze pollution when a certain economic level threshold is crossed. Therefore, the third hypothesis is proposed:

**Hypothesis** **3** **(H3).** *There is a threshold effect in the process of UIE improvement on haze governance, and the level of economic development can affect the position of the inflection point in the relationship between the two*.

**Figure 1:**
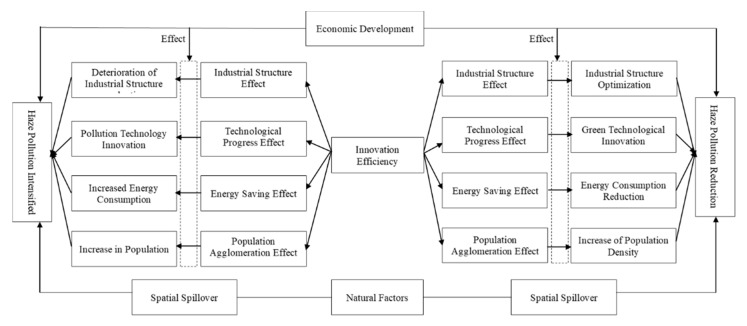
The mechanism of innovation efficiency on haze pollution.

Through the analysis of existing literature [2,18,19], we believe that innovation efficiency may affect the degree of haze pollution through the factors presented in Figure 1.

An improvement in UIE can promote the optimisation of the industrial structure in the following ways. First, the process of improving innovation efficiency guides the flow of innovation resources to more efficient sectors. It promotes further agglomeration of innovation resources, such as funds and capital for R&D, thereby enhancing the level of industrial technology and improving production. Second, such improvement continuously expands the potential boundary of production by improving production technology efficiency and production scale efficiency. These two ultimately promote an advanced industrial structure. A high proportion of secondary industries is an important reason for the aggravation of haze pollution [20,21]. Innovation promotes the transformation and upgrading of the industrial structure, which leads to the gradual phasing out of pollution-intensive secondary industries. Additionally, it actively develops modern service, technology-intensive, and tertiary industries [19]. With the optimisation of the industrial structure, the emission of air pollutants during the production process has decreased, and the haze pollution situation has gradually improved.

The improvement of UIE can greatly promote technological progress, especially the development of green innovative technologies, which can effectively reduce pollution emissions and alleviate haze pollution. Generally speaking, there are two main ways to control haze pollution: One is reusing air pollution emissions, and another is controlling the emission of air pollutants through advanced technologies [22]. Green technological progress, which usually comes from green innovation, refers to new technologies and products developed to reduce environmental damage [23]. This kind of progress promotes cleaner and more efficient production technology, which is conducive to promoting clean production and end governance to reduce pollutant emissions [12]. Technological advancements brought about by the improvement of UIE can improve energy efficiency and reduce the consumption of fossil fuels [24]. Most of the gas pollutants contained in haze come from fossil fuels [18]. Therefore, technological innovation can minimise the use of fossil fuels and other energy sources to reduce haze pollution.

With the improvement of UIE and the optimisation of industrial structure upgrading, an urban population inevitably gets attracted to the area, with R&D personnel as the core and various service industry employees to assist. This accelerates agglomeration, thereby exerting a certain influence on haze pollution. On the one hand, population gatherings have exacerbated haze pollution. An increase in the number of people leads to a higher demand for housing, household appliances, and consumption. There is also an increase in the discharge and incineration of domestic garbage, and the burning of heating energy during winter, inevitably raising the difficulty of haze governance. On the other hand, population agglomeration promotes haze governance to some extent. Population concentration means shorter commutes for urban residents, fewer cars in use per capita, and lower emissions of air pollutants in high-density urban areas [25]. In addition, it also leads to a concentration of enterprises and public facilities, which is conducive to the sharing of infrastructure, especially environmental pollution treatment facilities [26]. In summary, the fourth hypothesis is proposed:

**Hypothesis** **4** **(H4).** *UIE can affect haze pollution through the transmission of intermediary factors, such as industrial structure effects, technological progress effects, energy saving effects, and population agglomeration effects*.

## 3. Measurement Model and Index Description

### 3.1. Benchmark Model

The IPAT model proposed by Ehrlich and Holdren [27] is widely used for analysing the impact of human activities on the environment [28]. The basic equation of the model is *I* = *PAT*, where *I* stands for pollution, *P* is population, *A* is affluence level, and *T* is technical level. The IPAT model does not allow each influencing factor to change in non-monotonic or different proportions, so its application is greatly limited. To overcome this defect, Dietz and Rosa [29] developed the IPAT equation into the STIRPAT model:

*I_i_* = *a* × *P_i_^b^* × *A_i_^c^* × *T_i_^d^* × *e_i_* (1)

When the natural logarithm is taken on both sides, the equation becomes:

*lnI_i_* = *lna* + *b*(*lnP_i_*) + *c*(*lnA_i_*) + *d*(*lnT_i_*) + *ε_i_* (2)

The subscript *i* is the observation unit; *a* is a constant term; *b*, *c*, and *d* are the coefficients of each variable to be estimated; *e* is the error term; and *ε* is the logarithmic form of *e*. The advantage of the STIRPAT model is that it allows each coefficient to be used as the parameters can be estimated, and also allows each influencing factor to be decomposed.

Based on the STIRPAT model and Environmental Kuznets Curve (EKC) hypothesis [11], this study first constructed the following benchmark model for Hypothesis 1 to examine the impact of innovation efficiency on haze pollution:

*lnPM_it_* = *α*_0_ + *α*_1_*lnE_it_* + *α*_2_(*lnEit*)^2^ + *α*_3_*X_it_* + *ε_it_* (3)

### 3.2. Variable Selection and Data Source

The interpreted variable is haze pollution (*PM*), expressed as the annual average concentration of PM_2.5_. The global average annual value of the PM_2.5_ concentration was obtained from the International Geoscience Information Network Center of Columbia University. The ArcGIS software (Environmental Systems Research Institute, Redlands, CA, USA) was then used to analyse the average annual PM_2.5_ concentration data of all prefecture-level cities in China.

The core explanatory variable of this study is innovation efficiency (*E*), and its essence is an input-output ratio. We took the number of scientific research personnel and financial, science, and technology expenditures as innovation inputs [30,31]. The number of large patent applications and the number of scientific papers searched were taken as innovation outputs [32,33]. The number of scientific papers searched (pieces) consisted of Chinese and English papers, which were taken from the China Knowledge Network (CNKI) and the Web of Science (WOS) databases, respectively. The three major patent applications (cases) were retrieved according to the ‘Chinese Patent Full-text Database (Knowledge Edition)’, and the missing values of a few of the indicator data were completed with the linear interpolation method. The data envelopment analysis (DEA) method was used to calculate the innovation efficiency value of each city, and the specific processing was the same as Zhang et al. [34].

The threshold variable is the level of economic development (*Eco*); drawing on relevant research, we used the average brightness of night-time lights to represent the level of economic development [35,36]. Based on the EKC hypothesis, we also introduced the first and second terms of the economic variables in the model. Regarding the night-time light brightness data, we used the stable light data released by the National Oceanic and Atmospheric Administration (NOAA) website. Additionally, we selected the average light intensity data from 2012 to 2016. Due to the difficulty in converting the National Polar-Orbiting Partnership-Visible Infrared Imaging Radiometer Suite (NPP-VIIRS) data and the Defense Meteorological Satellite Program/Operational Line-Scan System (DMSP/OLS) data, this study selected 2012 as the base period. This processing technique is based on the methods of Shi et al. [37] and Chen et al. [38].

The intermediary variables include: (1) industrial structure (*sec*), measured by the proportion of the output value of the secondary industry to the gross domestic product (GDP); (2) technological progress (*tec*), measured by the number of patent licenses per 100 scientific research practitioners; (3) energy conservation (*es*), measured by the total annual liquefied petroleum gas supply; and (4) population agglomeration (*pop*) is expressed by the number of people per unit area [39,40]. Ma and Zhang [2] believed that the combustion of fossil fuels is an important source of haze pollution. The use of liquefied petroleum gas (LPG) reduces the combustion of fossil fuels, which is beneficial to haze governance.

We also included some factors related to haze pollution as control variables into the model: (1) fiscal expenditure (*pe*), measured by the number of local government budgetary fiscal expenditures; (2) transportation (*tri*), using a measure of the total annual passenger transport of public cars; and (3) the degree of opening to the outside world (*FDI*), measured by the amount of foreign direct investment actually used [41,42].

The data of the above variables were mainly obtained from the ‘China City Statistical Yearbook’ (2013–2017) and the EPS database (see Table 1 for details on the data sources). The collinearity test results of each variable indicate that there is no correlation between the explanatory variables (Table 1). Finally, we obtained panel data for a total of 1415 observations in 283 cities in China from 2012 to 2016. The correlation coefficient of each variable is shown in Table A1.

### 3.3. Spatial Weight Matrix

Haze pollution has a strong spatial correlation under the influence of natural activities, such as the atmosphere, and economic activities like industrial transfer. Therefore, the study of haze pollution needs to include a weight matrix reflecting the spatial relationship in the model. Based on the geography between cities, we constructed a geographical distance spatial weight matrix (W_1_) to reflect the influence of geographical factors on the spatial distribution characteristics of haze pollution. The element *w_ij_* of W_1_ represents the nearest highway mileage between cities *i* and *j*. In addition, we also obtained the economic geographic matrix W_2_, which reflects both urban economic and geographic information through MATLAB point multiplication. This was used for robustness testing. Referring to Shao et al. [4], let W_2_ = *ω*W_1_ + (1 − *ω*) W_3_, where *ω* represents the weight of the geographic distance spatial weight matrix, and the value is 0.5; W_3_ represents the weight matrix of the economic distance space, and its element, *w_ij_*, is the reciprocal of the absolute difference between the annual average per capita GDP of cities *i* and *j*.

### 3.4. Endogenous Issues

The two-way causal relationship between the explanatory and explained variables may lead to the existence of endogenous problems. The ‘Porter Hypothesis’ believes that the ultimate effect of environmental control imposed on companies is the promotion of continuous technological innovation. Therefore, innovation efficiency can affect haze pollution. In turn, strengthened environmental control and changes in environmental quality have also affected technological innovation. Serious endogenous issues will make the ordinary least squares (OLS) biased and inconsistent, and the problem of heteroscedasticity also exists. Thus, the estimation method will also be invalid. At this time, the lag item of the explanatory variable can be selected as a tool variable to solve the problem of invalid estimation, that is, using the 2SLS method for estimation. However, considering the spatial spill over effect of haze pollution, we further selected the GS2SLS estimation. This method selected all explanatory variables and their spatial hysteresis terms as instrumental variables, estimated the spatial panel model based on the 2SLS method [43], and simultaneously controlled the spatial correlation effect and endogenous problems in the model. When choosing the highest third-order spatial lag term as the instrument variable, the highest second-order spatial lag term was selected as the instrument variable in the robustness test.

## 4. Benchmark Regression Analysis

### 4.1. Benchmark Regression

Table 2 shows the GS2SLS estimation results of the benchmark model, and columns (1) and (2) present the estimation results of the fixed-effect and random-effect models considering only the core explanatory and basic variables of the STIRPAT model. Columns (3) and (4) add other control variables based on columns (1) and (2). The Hausman test of columns (1)–(4) in Table 2 all passed the significance level of 1%, which indicates that the fixed-effect model should be selected. The coefficients of the spatial hysteresis items of the haze pollution in Table 2 are significantly positive at the 1% level, indicating that haze pollution indeed has a spatial spill over effect, and haze pollution in adjacent regions will transfer to each other. This is because, on one hand, weather factors such as wind direction, temperature difference, and rainfall cause natural atmospheric flow. The degree of haze pollution in one area is closely related to the degree of haze pollution in geographically similar areas. On the other hand, human factors such as industrial transfer, cross-regional trade, and the externality of environmental policies have further strengthened the spatial correlation between regional haze governance and environmental air quality [44]. The regression results in columns (1)–(4) all show that the coefficient of the primary term of the core explanatory variable, innovation efficiency (*E*), is significantly positive, and that of the secondary term is significantly negative. There is a significant inverted ‘U’ relationship between innovation efficiency and haze pollution, and the latter presents a changing trend of first rising, and then falling with the improvement of innovation efficiency. According to column (3), we can also calculate the inflection point value of the inverted ‘U-shaped’ curve between innovation efficiency and haze pollution. We find that the innovation efficiency value of all cities is less than the inflection point value. During the research period, all cities in China are in the stage in which haze pollution increases with the improvement of innovation efficiency.

The first term coefficient of the economic factor (*Eco*) is significantly positive, and the second term coefficient is significantly negative. This is consistent with the EKC hypothesis. With the development of the urban economy, haze pollution first intensifies, and then alleviates. The development level was used as a threshold variable to explore whether there is a difference in the impact of UIE on haze pollution at different stages of economic development.

Population agglomeration (*pop*) has a significant positive effect on haze pollution. This is because as the urban population grows, the demand for and consumption of housing, household appliances, and motor vehicles increase, as do emission and burning of household waste and the burning of heating energy during winter. In addition, the real estate dust caused by population agglomeration, materials such as freon discharged by household appliances, automobile exhaust, and harmful substances from coal and garbage combustion are the foundations of haze formation. The coefficient of technical progress (*tec*) is negative. Technological advances have brought clean technologies and advanced environmental management methods to alleviate haze pollution. The coefficient of industrial structure (*sec*) is significantly positive, indicating that increasing the proportion of the secondary industry exacerbates haze pollution. Consistent with the conclusions of most studies on the relationship between industrial structure and environmental pollution, an increase in the proportion of secondary industries will lead to increased environmental pollution [20,21]. The coefficient of energy saving (*es*) is negative, and LPG is clean energy. The uses of clean energy can minimise the emission of atmospheric pollutants, thereby reducing haze pollution.

The coefficient sign of financial expenditure (*pe*) is positive, which may be because GDP growth was used as the main performance evaluation indicator in the promotion of Chinese local officials. To improve political performance, many local officials have excessively invested in construction and even introduced high pollution and high energy-consuming enterprises. This high-speed economic growth has brought serious environmental pollution problems. The coefficient of traffic (*tri*) is significantly positive, and the use of vehicles and other modes of transportation has exacerbated haze pollution. The coefficient of foreign investment (*FDI*) is not significant, and the impact of foreign direct investment on haze pollution cannot be determined. This indicates that neither the ‘pollution halo’ nor ‘pollution paradise’ hypotheses have been verified at the Chinese city level.

### 4.2. Robustness Test

We mainly adopted the method of replacing the explained variable, spatial weight matrix, and tool variable to test the robustness of the benchmark regression results. The specific approach is as follows. First, we used PM_10_ instead of PM_2.5_ to represent the air pollution variable for regression. The PM_10_ data were taken from the website of the Ministry of Ecology and Environment of China. This website has complete statistics on PM_10_ data for all prefecture-level cities in China since December 2014. Northern China begins burning coal for heating in December, and the data for December of the year is representative for studying haze pollution. Therefore, this study selected the monthly statistical data from December of 2014 to 2016 for testing; the geographical and economic distances nested weight matrix (W_2_) were used to replace the weight matrix (W_1_) from the previous regression. Based on the GS2SLS regression, the highest second-order spatial lag term was adopted to replace the highest third-order spatial lag term from the previous regression as the instrumental variable. Table 3 shows the regression results. The spatial lag term of haze pollution is still significant, and the inverted ‘U-shaped’ relationship between the core explanatory variable, UIE, and haze pollution is also significant. This shows that the baseline regression mentioned above has strong robustness.

## 5. Influence Difference and Transmission Mechanism

### 5.1. Regional Differences on the Impact of Innovation Efficiency on Haze pollution

To improve the level of innovation in Chinese cities, China began to implement the construction of an innovative city pilot project in 2008. Altogether, there are 78 pilot cities in China. Lhasa, Shihezi, and four other cities were not included due to a lack of statistical data. Therefore, a total of 74 innovative pilot cities were included in the sample. First, a difference analysis was conducted on the data for innovative pilot and non-pilot cities. The results show that there are significant differences in the mean values of PM_2.5_ concentration and innovation efficiency between the two samples, and the grouping is effective.

**Figure 2:**
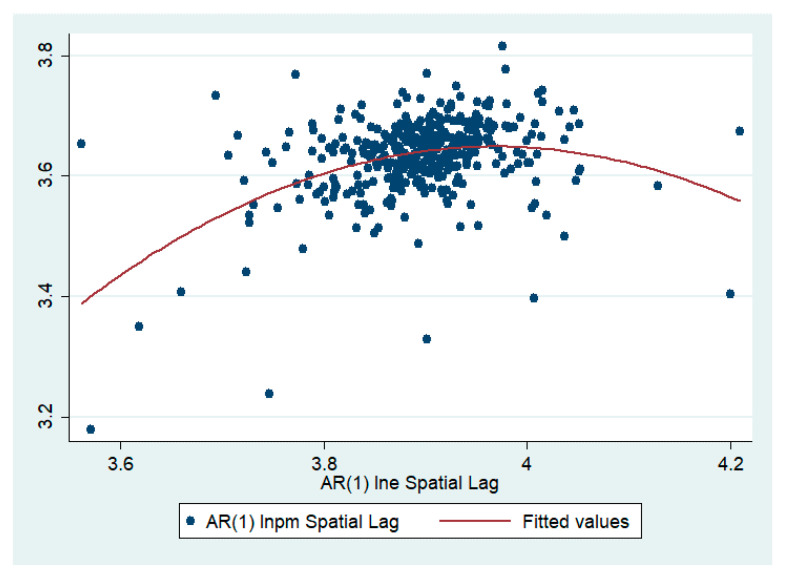
Nonlinear scatter fit graph of innovation efficiency and haze pollution.

Second, a regression of the pilot cities was carried out. The regression results are listed in Table 4. The relationship between innovation efficiency and haze pollution shows an inverted ‘U’ type relationship, but the coefficient is not significant. To further illustrate that the regression results are also meaningful to a certain extent, we drew a scatter plot with the haze pollution of the pilot city as the *y*-axis and the innovation efficiency as the *x*-axis (Figure 2). We find that the fitting curve of the two showed an inverted ‘U’ shape. Therefore, the regression conclusion is valid: In pilot cities, the increase in initial innovation efficiency leads to increased haze pollution. When innovation efficiency increases to a certain level, haze pollution gradually decreases.

We also conducted a regression for non-pilot cities. A significant difference is that the primary term coefficient of the core explanatory variable, innovation efficiency (*E*), is significantly negative, while the quadratic term coefficient is significantly positive. Moreover, the relationship between innovation efficiency and haze pollution presents a positive ‘U’ shape. Although in the short term, non-pilot cities are at the stage of haze pollution reduction with the improvement of innovation efficiency, this scenario is not sustainable. Once the innovation efficiency value exceeds the inflection point value, the improvement of innovation efficiency will eventually aggravate haze pollution.

Comparing the regression results of the pilot and non-pilot cities, as the efficiency of innovation increases, haze pollution in pilot cities first increases then alleviates, while haze pollution in non-pilot cities first alleviates then finally increases. Hypothesis 2 was established, which provides some policy recommendations for China’s haze governance: The current development method of building innovative cities and countries implemented in China is environmentally friendly. Although improving the efficiency of innovation will bring some short-term pollution problems, increased efficiency can alleviate haze pollution in the long run. China should summarise the existing pilot experience while expanding the range of pilot innovative cities in an orderly manner.

### 5.2. The Stage Difference in the Impact of Innovation Efficiency on Haze Pollution

#### 5.2.1. Threshold Model Setting

The benchmark regression results above show that there is a non-linear relationship between innovation efficiency and haze pollution. Here, we used a threshold regression model to further explore this relationship. A threshold regression tests whether a sample group parameter divided according to the threshold value is significantly different [45]. The threshold regression model developed by Hansen [46] can divide the data interval endogenously according to the characteristics of the data itself, avoiding the arbitrariness of artificially dividing the sample interval. Therefore, this research utilised the above threshold regression model, and used the economic development level as the threshold variable, combined with the logarithmic form in the benchmark model, to set the following model:

*ln PM_it_* = *φ*_0_ + *φ*_1_*ln E_it_ ▪I*(*ln Eco_it_* ≤ *γ*_1_) + *φ*_2_*ln E_it_ ▪I(ln Eco_it_* > *γ*_1_) + *φ*_3_*ln Eco_it_* + *φ*_4_*X_it_* + *ε_it_* (4)

*I* (*▪*) represents an indicative function. When the expression in the brackets is true, the value is 1; when it is false, the value is 0. *φ*_0_ is a constant, *E_it_* is the core explanatory variable, and *Eco_it_* is the threshold variable. *X_it_* represents the control variable, and *ε_it_* is a random disturbance term. When ln*Eco_it_* ≤ *γ*_1_, the coefficient of the core explanatory variable ln*E_it_* is *φ*_1_; when ln*Eco_it_* > *γ*_1_, the coefficient of the core explanatory variable ln*E_it_* is *φ*_2_, and the similarities and differences between *φ*_1_ and *φ*_2_ are our focus.

The above model is applicable to the case of a single threshold, and the following model is applicable to the case of a double threshold:

*ln PM_it_* = *φ*_0_ + *φ*_1_*ln E_it_ ▪I(ln Eco_it_* ≤ *γ*_1_) + *φ*_2_*ln E_it_ ▪I(γ*_1_ <*ln Eco_it_* < *γ*_2_) + *φ*_3_*ln E_it_ ▪I(ln Eco_it_* > *γ*_2_) + *φ*_4_*ln Eco_it_* + *φ*_5_*X_it_* + *ε_it_* (5)

Considering the spatial spill over effect of haze pollution and possible endogenous problems in the model, during threshold regression, the spatial lag term of haze pollution obtained by the GS2SLS method, and the first-order spatial lag term of each variable were introduced to replace the original variable.

#### 5.2.2. Threshold Regression Analysis

Innovative pilot and non-pilot cities have significant differences in terms of innovation efficiency, but there is no significant difference in the level of economic development. There are obvious differences in economic development among the eastern, central, and western regions of China (there are 115 cities in the eastern region, including Hebei, Liaoning, Jiangsu, Zhejiang, Fujian, Shandong, Guangdong, Guangxi, Hainan, Beijing, Tianjin, Shanghai, 12 provinces, autonomous regions, and municipalities. There are 109 cities in the central region, including Shanxi and Jilin, Heilongjiang, Anhui, Jiangxi, Henan, Hubei, Hunan, Inner Mongolia, nine provinces and autonomous regions. There are 59 cities in the western region, including Sichuan, Guizhou, Yunnan, Shaanxi, Gansu, Ningxia, Qinghai, Xinjiang, eight provinces and autonomous regions). Thus, we in turn conducted a single threshold assumption, double threshold hypothesis, and triple threshold hypothesis test for the overall sample, as well as for the eastern, central, and western cities; the test results are shown in Table 5. Overall, the whole sample and the western cities accepted the assumption of the double threshold model at a significance level of 10%, while the eastern and central cities accepted the that of the single threshold model at the significance levels of 1% and 10%, respectively. Therefore, Hypothesis 3 is valid. As such, this study adopted a double-threshold model regression for the whole sample and the western cities, and a single-threshold model regression for the eastern and central cities (Table 6).

The regression results of the panel threshold model are shown in Table 7 and Table 8. The numbers in parentheses below are the corresponding night-time light brightness values. When the economic level is below the first threshold value of −0.984 (corresponding to the night-time light brightness of 0.374), the elasticity coefficient of innovation efficiency to haze pollution is −0.249. When the economic level is between two thresholds, the elasticity coefficient of innovation efficiency to haze pollution is −0.269. When the economic development level exceeds the second threshold of −0.507 (0.602), the elasticity coefficient of innovation efficiency on haze pollution is −0.276. This indicates that cities in China have initially achieved the ‘win-win’ goal of improving the level of innovation and haze governance. Improvement of the overall innovation efficiency of Chinese cities can effectively reduce haze pollution. The higher the level of urban economic development, the clearer this mitigation effect is. Similarly, after the development level exceeds the unique threshold of −0.945 (0.389), the effect of improved innovation efficiency on haze governance becomes increasingly evident in eastern cities.

In the central region, when economic level is below the single threshold value of −0.424 (0.654), the elastic coefficient of innovation efficiency to haze pollution is 0.420, and it is 0.431 after crossing the threshold. With improvement in the economic development level, the improvement of innovation efficiency only aggravates haze pollution. This shows that the central region is still in a transition stage of development, although the innovation technology level has risen in recent years. However, with the industrial gradient and potential energy difference between the eastern and central regions, the former has transferred backward production capacity and environmentally polluting industries to the latter, which has led to increasing innovation efficiency and haze pollution coexisting in the study period.

In cities in the western region, when the first threshold −1.271 (0.281) is not exceeded, the elasticity coefficient of innovation efficiency on haze pollution is −2.664. When the economic level is between the two thresholds, the elasticity coefficient of innovation efficiency on haze pollution is −0.269. When the economic development level crosses the second threshold of −1.037 (0.355), the elasticity coefficient of innovation efficiency for haze pollution is −0.160. In western cities, as the economic development level and innovation efficiency increases, the mitigation effect of haze pollution weakens. This is because the western region of China is economically underdeveloped, and the economic development gap continues to expand. To eliminate this gap, R&D investment in the western region has gradually concentrated on improving productivity, and the effectiveness of innovation efficiency in suppressing haze pollution is becoming increasingly slow.

### 5.3. The Influence Mechanism of Innovation Efficiency Improvement on Haze Pollution

As can be seen from the foregoing, innovation efficiency may affect haze pollution in four ways: an industrial structure effect, energy saving effect, technological progress effect, and population agglomeration effect. Here, a mediation effect model comprising the following three regression equations was constructed to identify and test the above conduction pathways:

*ln PM_it_* = *θ*_0_ + *θ*_1_*lnE_it_* + *θ*_2_ (*ln E_it_*)^2^ + *θ*_3_*Υ_it_* + *ξ_it_* (6)

*D_it_* = *β*_0_ + *β*_1_*lnE_it_* + *β*_2_ (*ln E_it_*)^2^ + *β*_3_*Υ_it_* + *μ_it_* (7)

*ln PM_it_* = *γ*_0_ + *γ*_1_*lnE_it_* + *γ*_2_ (*ln E_it_*)^2^ + *γ*_3_*Υ_it_* + *γ*_4_*D_it_* + *τ_it_* (8)

*Y* is a vector set composed of control variables; *D* is a possible intermediary variable, including industrial structure (ln*sec*), energy saving (ln*es*), technological progress (ln*tec*), and population agglomeration (ln*pop*); and *E* and *PM* are innovation efficiency and PM_2.5_ concentration, respectively. According to the principle of the intermediary effect model [6], if the coefficient *θ*_1_ or *θ*_2_, *β*_1_ or *β*_2_, and *γ*_4_ are all significant, and the coefficients *γ*_1_ and *γ*_2_ become smaller or significantly lower than *θ*_1_ and *θ*_2_, then there is an intermediary effect.

According to the test results of the intermediary effect (Table 8), when technological progress is regarded as an intermediary variable, the coefficients of the first and second terms of the UIE in (8) are larger than the corresponding coefficients in (6). When energy saving is regarded as an intermediary variable, the coefficients of the first and second terms of UIE in (7) are not significant. Therefore, it can be judged that the technological progress effect and energy saving effect are not the transmission pathways of innovation efficiency affecting haze pollution. For industrial structure and population agglomeration, the coefficients of the first and second terms of innovation efficiency in Equations (6) and (7) are significant, and the corresponding coefficients in Equation (8) are reduced compared with Equation (6). Further, the industrial structure and population agglomeration meet the criteria of intermediary variables. Therefore, in the effect of innovation efficiency on haze pollution, industrial structure and population agglomeration effects have an intermediary effect. Thus, Hypothesis 4 is true. It can be observed that the process of UIE is improving, and the haze reduction effect of industrial structure optimisation and population concentration has not been effectively exerted, which is an important reason for the increase of haze pollution in China.

We also conducted the same mediating effect test for samples from different regions. The variables ln*E* and (ln*E*)^2^ in the basic regression of samples from innovative pilot cities did not pass the significance test; they did not meet the requirement that the mediating effect model test principle coefficient, *θ*_1_ or *θ*_2_, be significant. Therefore, only non-innovative pilot cities were tested in the next step. The results show that the mediating effects of energy saving, technological progress, and population agglomeration are not established, and innovation efficiency affects haze pollution through the industrial structure effect. By comparing the results in Table 9 and Table 10, it can be found that under the combined effect of industrial structure and population agglomeration effects, the relationship between innovation efficiency and haze pollution is an inverted ‘U-shaped’ curve. In the long run, the improvement of innovation efficiency will ultimately reduce haze pollution. However, if we only focus on optimising the industrial structure without giving full play to the population agglomeration effect, there will be a positive ‘U-shaped’ curve relationship between innovation efficiency and haze pollution. Further, an improvement in innovation efficiency will eventually lead to the intensification of haze pollution. The innovation-oriented city pilot policy can promote the concentration of talent, stimulate enterprise investment, optimise the innovation environment, and exert a positive impact on urban innovation and haze governance. Thus, on one hand, we should vigorously develop the policy of building innovative cities and promote its role in achieving the ‘win-win’ goal of improving innovation efficiency and mitigating haze pollution. On the other hand, attention should be paid to the fact that merely improving innovation efficiency is not conducive to realising the long-term haze reduction target in the process of implementing this policy. The focus should be placed on the optimisation of the industrial structure and population concentration.

## 6. Conclusions

Taking the panel data for 283 prefectures-level cities in China from 2012 to 2016 as samples, this study systematically investigated the relationship between innovation efficiency and haze pollution by adopting the GS2SLS method. We further discussed the difference in the impact of innovation efficiency on haze pollution at different stages of economic development and its transmission mechanism. Based on our analysis, we reached the following conclusions:

(1) After considering the spatial spill over effect of haze pollution and controlling endogeneity, the relationship between innovation efficiency and haze pollution in China presents an inverted ‘U’ shape. With the improvement of innovation efficiency, the degree of haze pollution will be continuously reduced. However, the innovation efficiency of Chinese cities in the research period has not reached the inflection point of the inverted ‘U-shaped’ curve, which is located on the left side of the curve. In different economic development ranges, the impact of innovation efficiency on haze pollution is not the same. There is a double threshold effect; as the economic level crosses the first threshold of −0.984 (night-time light brightness value is 0.374) and the second threshold of −0.507 (night-time light brightness value is 0.602), the effect of innovation efficiency on haze pollution strengthened.

(2) In terms of regional differences, the innovation efficiency and haze pollution of innovative pilot cities show an inverted ‘U’ relationship, and the shape of the relationship curve in non-pilot cities is reversed to a positive ‘U’ type. In the long run, the improvement of innovation efficiency of innovative cities in haze governance is better than that of non-innovative pilot cities. In the threshold test, the central region shows that haze pollution is continuously aggravated by the improvement of innovation efficiency as the economic development level crosses the threshold. The reason for this is that the central region is still in the transitional stage of economic transformation and development. In recent years, the level of innovation and technology has constantly improved, but at the same time, environmental polluting industries in the eastern region have continuously transferred. This led to the co-existence of innovation efficiency improvement and haze pollution intensification in the research period. In western China, as the level of economic development crosses the threshold, the inhibiting effect of innovation efficiency on haze pollution is weakened. Due to the backward economic development level and widening economic gap in the western region, R&D investment in this region has been constantly concentrated in the direction of production, ignoring environmental benefits.

(3) The improvement of innovation efficiency can affect haze pollution through the transmission of two intermediary factors: the industrial structure effect and population agglomeration effect. In the process of improving UIE, the optimisation of the industrial structure and the effect of reducing population agglomeration have not been effectively exerted. This is an important reason for the increase of haze pollution in China. Further, comparing the differences between China as a whole and its various regions, it can be observed that under the combined effect of the industrial structure and population agglomeration effects, the relationship between innovation efficiency and haze pollution is an inverted ‘U-shaped’ curve. However, if we only focus on optimising the industrial structure without giving full play to the population agglomeration effect, there will be a positive ‘U-shaped’ curve relationship between innovation efficiency and haze pollution. Additionally, improvement in innovation efficiency will eventually lead to the intensification of haze pollution.

On the basis of our findings, we propose the following policy recommendations for haze pollution governance:

(1) China should take measures to comprehensively improve the efficiency of innovation. These can include further increasing government investment in science and technology, or accelerating the establishment of an effective mechanism for monitoring scientific research results. Crossing the inflection point as soon as possible should be considered to realise the haze reduction effect of improving innovation efficiency.

(2) China should continue to adhere to the strategy of innovation-driven economic development, and through mutual promotion and continuous improvement of the level of innovation and economic development, form a strong joint force for haze governance. In the process of innovation-driven economic development, innovation should not only aim at economic benefits, but also pay attention to green technology innovation. It should avoid the introduction of high-pollution technologies so as to reduce haze pollution and achieve high-quality economic development.

(3) China should further expand the trial of innovative cities. Through innovative city pilot policies, cities can attract high-tech industries and high-end talent to promote the optimisation and upgrading of their industrial structure and population agglomeration. By comprehensively utilising the effect of industrial structure and population agglomeration, we can ensure that innovation efficiency plays a role in reducing haze in the long run.

According to the study results presented in this paper, the DEA model has strengthened the comparability and distinction between DMUs. On the premise of the full respect for objectivity, the influence of preference of decision makers is considered properly which is feasible to evaluate the core explanatory variable innovation efficiency. However, regarding the research on innovation efficiency at the city level of China’s prefecture-level cities, because the statistics at the prefecture-level cities in China are incomplete, some innovation indicators do not have relevant statistical data, which limits this research. In fact, urban innovation efficiency reflects the relative effect of the allocation of innovative resources in each city, indicating the strength of the ability of each city to allocate innovative resources, rather than the true value of urban innovation efficiency. This relativity can directly depict the differences of the efficiency of the allocation of innovative resources in various cities. At the same time, we believe that with the further advancement of follow-up studies and the application of big data, the selection of new core explanatory variables for urban innovation efficiency will be more scientific.

## Figures and Tables

**Table 1:** Variable description and data sources.

Variable Type	Variable Name	Indicator	Data Sources
Explained variable	Haze pollution	PM_2.5_ annual average concentration	Columbia University International Earth Science Information Network (https://beta.sedac.ciesin.columbia.edu/)
Core explanatory variable	Innovation efficiency	Input-output ratio of innovative behaviour	Calculated by DEA method
Threshold variable	The level of economic development	Average night light brightness	NOAA WEBSITE (https://ngdc.noaa.gov/eog/index.html)
Intermediary variable	Industrial structure	The output value of secondary industry accounts for the proportion of GDP	China City Statistical Yearbook (2013–2017)
	Technical progress	The number of patent authorisations per hundred scientific research practitioners	
	Energy saving	Annual LPG gas supply	EPS DATABASE
	Population agglomeration	Population per unit area	China City Statistical Yearbook (2013–2017)
Control variable	Fiscal expenditure	Local government general budget expenditure	EPS DATABASE
	Transportation	Total passenger transport of public motor vehicles	China City Statistical Yearbook (2013–2017)
	Trade openness	The amount of foreign capital actually utilised	

**Table 2:** Benchmark regression.

Variable	(1)	(2)	(3)	(4)
	FE	RE	FE	RE
*W*_1_ * ln*PM*	1.098 *** (0.361)	1.044 *** (0.075)	1.082 *** (0.348)	0.925 *** (0.075)
ln*E*	0.733 *** (0.093)	1.322 *** (0.050)	0.371 *** (0.109)	0.501 *** (0.101)
(ln*E*)^2^	−0.084 *** (0.013)	−0.166 *** (0.008)	−0.034 ** (0.015)	−0.054 *** (0.014)
ln*Eco*	0.062 *** (0.010)	0.066 *** (0.009)	0.046 *** (0.010)	0.039 *** (0.010)
(ln*Eco*)^2^	−0.025 *** (0.004)	−0.025 *** (0.004)	−0.025 *** (0.004)	−0.027 *** (0.004)
ln*pop*	0.030 *** (0.008)	0.047 *** (0.008)	0.023 *** (0.008)	0.033 *** (0.008)
ln*tec*	−0.071 *** (0.011)	−0.059 *** (0.010)	−0.073 *** (0.011)	−0.057 *** (0.010)
ln*sec*			0.155 *** (0.036)	0.170 *** (0.033)
ln*es*			−0.009 ** (0.004)	−0.008 ** (0.004)
ln*pe*			0.027 * (0.016)	0.045 *** (0.015)
ln*tri*			0.041 *** (0.014)	0.047 *** (0.013)
ln*FDI*			−0.004 (0.006)	0.001 (0.006)
Adjust R^2^	0.980	0.980	0.981	0.981
Wald test (*p*)	293.861 (0.000)	5271.420 (0.000)	358.234 (0.000)	5775.189 (0.000)
Hausman test (*p*)	94.820 (0.000)		117.609 (0.000)	

Note: ***, **, and * represent the significance levels of 1%, 5%, and 10%, respectively; the values in parentheses below the coefficients are their standard errors; FE and RE represent fixed-effect models and random-effect models, respectively; this also applies to the following tables.

**Table 3:** Robustness test.

Variable	Replace Explained Variables	Replace Spatial Weight Matrix	Replace Tool Variables
*W* * ln*PM*	0.768 *** (0.188)	0.146 *** (0.036)	0.923 *** (0.076)
ln*E*	0.135 * (0.227)	0.614 *** (0.103)	0.504 *** (0.101)
(ln*E*)^2^	−0.005 * (0.031)	−0.068 *** (0.015)	−0.054 *** (0.014)
ln*Eco*	−0.017 (0.012)	0.034 *** (0.010)	0.039 *** (0.010)
(ln*Eco*)^2^	−0.016 *** (0.006)	−0.029 *** (0.004)	−0.027 *** (0.004)
ln*pop*	0.024 ** (0.012)	0.040 *** (0.008)	0.033 *** (0.008)
ln*tec*	−0.041 *** (0.015)	−0.045 *** (0.010)	−0.057 *** (0.010)
ln*sec*	0.272 *** (0.054)	0.201 *** (0.034)	0.170 *** (0.033)
ln*es*	−0.009 ** (0.005)	−0.007 * (0.004)	−0.008 ** (0.004)
ln*pe*	−0.027 (0.027)	0.052 *** (0.015)	0.045 *** (0.014)
ln*tri*	0.035 * (0.019)	0.043 *** (0.014)	0.047 *** (0.013)
ln*FDI*	0.003 (0.008)	0.012 * (0.006)	0.001 (0.006)
Adjust R^2^	0.650	0.980	0.981
Wald test (*p*)	89.025 (0.000)	4863.616 (0.000)	5767.449 (0.000)

Note: ***, **, and * represent the significance levels of 1%, 5%, and 10%, respectively. Due to space limitations, and based on the Hausman test results, this table only reports the estimation results based on a more desirable random effect model. The following tables are the same. W represents W_1_ in the method of replacing explained variables and instrumental variables, and W represents W_2_ in the method of replacing space weight matrix.

**Table 4:** Grouped inspection results of innovative cities and non-innovative cities.

Group	ln*PM*	ln*E*	(ln*E*)^2^
Non-policy pilot cities (mean)	3.420	3.482	12.389
Innovative pilot cities (mean)	3.614	3.896	15.506
Mean test (t value)	−6.449 ***	−12.894 ***	−13.461 ***

Note: The mean difference test is to test the t value; *** indicate significance at the levels of 1%.

**Table 5:** Sample regression by region.

Variable	Innovative Pilot City	Non-Pilot City
*W*_1_ * ln*PM*	0.780 * (0.408)	1.066 *** (0.246)
ln*E*	0.334 (0.299)	−0.601 *** (0.214)
(ln*E*)^2^	−0.025 (0.040)	0.079 *** (0.030)
ln*Eco*	0.007 (0.024)	0.002 (0.015)
(ln*Eco*)^2^	−0.025 ** (0.013)	−0.055 *** (0.006)
ln*pop*	0.064 *** (0.019)	0.045 ** (0.018)
ln*tec*	0.041 (0.026)	0.028 * (0.015)
ln*sec*	0.496 *** (0.103)	0.064 (0.056)
ln*es*	−0.032 *** (0.009)	−0.002 (0.005)
ln*pe*	0.175 *** (0.054)	0.050 (0.032)
ln*tri*	−0.106 *** (0.037)	0.054 * (0.020)
ln*FDI*	0.058 *** (0.020)	0.003 (0.001)
Adjust R^2^	0.538	0.648
Wald test (*p*)	186.156 (0.000)	289.628 (0.000)

Note: ***, **, and * represent the significance levels of 1%, 5%, and 10%, respectively.

**Table 6:** Threshold effect test.

Group	All the Cities	Eastern Cities	Central Cities	Western Cities
Single threshold	37.920 ***	29.510 ***	13.850 *	17.510 **
Double threshold	14.310 *	16.590	7.480	16.020 *
Three thresholds	11.690			7.820

Note: The data in the table is the F statistic corresponding to the threshold test, and ***, **, and * indicate significance at the levels of 1%, 5%, and 10%, respectively.

**Table 7:** Threshold and confidence interval estimation.

Group	All the Cities	Eastern Cities	Central Cities	Western Cities
Threshold estimate 1	−0.984	−0.945	−0.424	−1.271
95% confidence interval	[−1.008, −0.974]	[−1.016, −0.847]	[−0.438, −0.423]	[−1.295, −1.144]
Night light brightness 1	0.374	0.389	0.654	0.281
Threshold estimate 2	−0.507			−1.037
95% confidence interval	[−0.524, −0.505]			[−1.048, −1.034]
Night light brightness 2	0.602			0.355

**Table 8:** Parameter estimation results of threshold regression model.

Variable	All the Cities	Eastern Cities	Central Cities	Western Cities
ln*E* (ln*Eco* ≤ *γ*_1_)	−0.249 *** (0.060)	−0.229 ** (0.090)	0.420 *** (0.117)	−2.646 *** (0.722)
ln*E* (*γ*_1_ < ln*Eco* ≤ *γ*_2_)	−0.269 *** (0.059)	−0.262 *** (0.090)	0.431 *** (0.118)	−0.139 (0.258)
ln*E* (ln*Eco* > *γ*_2_)	−0.276 *** (0.060)			−0.160 (0.259)
*W*_1_ * ln*PM*	2.241 *** (0.054)	2.105 *** (0.071)	2.096 *** (0.090)	2.552 *** (0.192)
ln*Eco*	−0.195 *** (0.035)	−0.149 *** (0.054)	−0.272 *** (0.060)	0.049 (0.137)
ln*pop*	−0.014 (0.032)	−0.061 (0.144)	−0.511 *** (0.128)	−0.050 (0.066)
ln*tec*	0.365 *** (0.045)	0.496 *** (0.079)	0.175 ** (0.087)	0.503 *** (0.125)
ln*sec*	−1.319 *** (0.145)	−0.583 ** (0.226)	−0.851 *** (0.285)	−1.699 *** (0.476)
ln*es*	0.196 *** (0.026)	0.174 *** (0.048)	0.207 *** (0.044)	0.011 (0.057)
ln*pe*	−0.534 *** (0.079)	−0.550 *** (0.126)	−0.226 (0.199)	−1.265 *** (0.256)
ln*tri*	0.235 *** (0.071)	0.172 * (0.087)	0.179 (0.135)	−0.109 (0.288)
ln*FDI*	0.090 *** (0.023)	0.094 *** (0.029)	0.060 (0.047)	−0.061 (0.082)

Note: ***, **, and * represent the significance levels of 1%, 5%, and 10%, respectively.

**Table 9:** Mediation effect test.

**Variable**	***D* = ln*sec***			***D* = ln*es***		
	**(6)**	**(7)**	**(8)**	**(6)**	**(7)**	**(8)**
ln*E*	0.554 *** (0.102)	1.185 *** (0.077)	0.370 *** (0.109)	0.373 *** (0.110)	−0.186 (0.800)	0.370 *** (0.109)
(ln*E*)^2^	−0.061 *** (0.014)	−0.175 *** (0.011)	−0.033 ** (0.015)	−0.034 ** (0.015)	0.066 (0.113)	−0.033 ** (0.015)
*D*			0.155 *** (0.035)			−0.009 ** (0.004)
**Variable**	***D* = ln*tec***			***D* = ln*pop***		
	**(6)**	**(7)**	**(8)**	**(6)**	**(7)**	**(8)**
ln*E*	0.282 ** (0.110)	1.167 *** (0.283)	0.370 *** (0.109)	0.397 *** (0.109)	1.082 *** (0.365)	0.370 *** (0.109)
(ln*E*)^2^	−0.028 * (0.016)	−0.062 (0.040)	−0.033 ** (0.015)	−0.037 ** (0.015)	−0.146 *** (0.051)	−0.033 ** (0.015)
*D*			−0.073 *** (0.010)			0.023 *** (0.008)

Note: ***, **, and * represent the significance levels of 1%, 5%, and 10%, respectively.

**Table 10:** Non-pilot city intermediary effect test.

**Variable**	***D* = ln*sec***			***D* = ln*es***		
	**(6)**	**(7)**	**(8)**	**(6)**	**(7)**	**(8)**
ln*E*	−0.622 *** (0.213)	−0.264 ** (0.126)	−0.601 *** (0.214)	−0.602 *** (0.214)	−0.765 (1.400)	−0.601 *** (0.214)
(ln*E*)^2^	0.081 *** (0.030)	0.024 (0.018)	0.079 *** (0.030)	0.079 *** (0.030)	0.166 (0.194)	0.079 *** (0.030)
*D*			0.064 (0.056)			−0.002 (0.005)
**Variable**	***D* = ln*tec***			***D* = ln*pop***		
	**(6)**	**(7)**	**(8)**	**(6)**	**(7)**	**(8)**
ln*E*	−0.551 ** (0.212)	1.754 *** (0.452)	−0.601 *** (0.214)	−0.602 *** (0.214)	−0.045 (0.389)	−0.601 *** (0.214)
(ln*E*)^2^	0.076 ** (0.030)	−0.119 * (0.063)	0.079 *** (0.030)	0.080 *** (0.030)	0.026 (0.054)	0.079 *** (0.030)
*D*			0.028 * (0.015)			0.045 ** (0.018)

Note: ***, **, and * represent the significance levels of 1%, 5%, and 10%, respectively.

## Appendix A

**Table A1:** Correlation coefficient of each variable.

	ln*PM*	ln*E*	(ln*E*)^2^	ln*Eco*	(ln*Eco*)^2^	ln*pop*	ln*tec*	ln*sec*	ln*es*	ln*pe*	ln*tri*	ln*fdi*
**ln*PM***	1											
**ln*E***	0.237 ***	1										
**(ln*E*)^2^**	0.237 ***	0.995 ***	1									
**ln*Eco***	0.396 ***	0.446 ***	0.453 ***	1								
**(ln*Eco*)^2^**	−0.389 ***	−0.116 ***	−0.108 ***	−0.513 ***	1							
**ln*pop***	0.334 ***	0.109 ***	0.110 ***	0.141 ***	−0.101 ***	1						
**ln*tec***	0.298 ***	0.539 ***	0.528 ***	0.451 ***	−0.222 ***	0.0727 ***	1					
**ln*sec***	0.140 ***	−0.0694 ***	−0.0750 ***	0.171 ***	−0.277 ***	−0.00620	0.146 ***	1				
**ln*es***	0.178 ***	0.240 ***	0.248 ***	0.318 ***	−0.0351	0.168 ***	0.212 ***	−0.110 ***	1			
**ln*pe***	0.309 ***	0.304 ***	0.317 ***	0.446 ***	−0.0314	0.395 ***	0.169 ***	−0.138 ***	0.374 ***	1		
**ln*tri***	0.363 ***	0.206 ***	0.210 ***	0.412 ***	−0.226 ***	0.340 ***	0.160 ***	0.134 ***	0.236 ***	0.652 ***	1	
**ln*fdi***	0.467 ***	0.305 ***	0.315 ***	0.553 ***	−0.235 ***	0.266 ***	0.280 ***	0.0857 ***	0.392 ***	0.620 ***	0.501 ***	1

Note: *** represent the significance levels of 1%.
